# Cortical Amyloid Beta in Cognitively Normal Elderly Adults is Associated with Decreased Network Efficiency within the Cerebro-Cerebellar System

**DOI:** 10.3389/fnagi.2014.00052

**Published:** 2014-03-18

**Authors:** Stefanie C. Steininger, Xinyang Liu, Anton Gietl, Michael Wyss, Simon Schreiner, Esmeralda Gruber, Valerie Treyer, Andrea Kälin, Sandra Leh, Alfred Buck, Roger M. Nitsch, Klaas P. Prüssmann, Christoph Hock, Paul G. Unschuld

**Affiliations:** ^1^Division of Psychiatry Research and Psychogeriatric Medicine, University of Zürich, Zürich, Switzerland; ^2^Department of Radiology, Brigham and Women’s Hospital, Harvard Medical School, Boston, MA, USA; ^3^Institute for Biomedical Engineering, University of Zürich, ETH Zürich, Zürich, Switzerland; ^4^Division of Nuclear Medicine, University of Zürich, Zürich, Switzerland

**Keywords:** cerebellum, fMRI BOLD, functional connectivity, Alzheimer disease, amyloid beta-peptides, aging neuroscience

## Abstract

**Background:** Deposition of cortical amyloid beta (Aβ) is a correlate of aging and a risk factor for Alzheimer disease (AD). While several higher order cognitive processes involve functional interactions between cortex and cerebellum, this study aims to investigate effects of cortical Aβ deposition on coupling within the cerebro-cerebellar system.

**Methods:** We included 15 healthy elderly subjects with normal cognitive performance as assessed by neuropsychological testing. Cortical Aβ was quantified using (11)carbon-labeled Pittsburgh compound B positron-emission-tomography late frame signals. Volumes of brain structures were assessed by applying an automated parcelation algorithm to three dimensional magnetization-prepared rapid gradient-echo T1-weighted images. Basal functional network activity within the cerebro-cerebellar system was assessed using blood-oxygen-level dependent resting state functional magnetic resonance imaging at the high field strength of 7 T for measuring coupling between cerebellar seeds and cerebral gray matter. A bivariate regression approach was applied for identification of brain regions with significant effects of individual cortical Aβ load on coupling.

**Results:** Consistent with earlier reports, a significant degree of positive and negative coupling could be observed between cerebellar seeds and cerebral voxels. Significant positive effects of cortical Aβ load on cerebro-cerebellar coupling resulted for cerebral brain regions located in inferior temporal lobe, prefrontal cortex, hippocampus, parahippocampal gyrus, and thalamus.

**Conclusion:** Our findings indicate that brain amyloidosis in cognitively normal elderly subjects is associated with decreased network efficiency within the cerebro-cerebellar system. While the identified cerebral regions are consistent with established patterns of increased sensitivity for Aβ-associated neurodegeneration, additional studies are needed to elucidate the relationship between dysfunction of the cerebro-cerebellar system and risk for AD.

## Introduction

Deposition of cortical amyloid beta (Aβ) is a correlate of aging and a risk factor for pathological cognitive decline due to Alzheimer disease (AD) (Sperling et al., 2011; Villemagne et al., 2011, 2013). Cortical Aβ affects various aspects of brain functioning (Morris et al., 2009; Storandt et al., 2009) and may reflect an early stage of AD development (Mormino et al., 2012).

While there is significant variation in elderly persons both regarding the degree of brain amyloidosis (Price et al., 2009; Mormino et al., 2012) as well as cognitive performance levels (Christensen et al., 1999), the term cognitive reserve is used to describe individual resilience against aging-associated decline of brain functionality by maintaining normal cognitive performance despite manifesting cerebral pathology (Stern, 2009). Besides brain amyloidosis, several other processes including tauopathy and inflammation have been suggested to contribute to aging-related disturbances of brain functionality (Gruart et al., 2008; Krstic and Knuesel, 2013) and individual neuronal properties may constitute resilience against cognitive decline in this context (Steffener and Stern, 2012). Functional connectivity at rest has been shown to directly relate to cognitive reserve and has been shown capable of measuring brain amyloidosis-associated functional deficits prior to development of cognitive deficits (Hedden et al., 2009; Mormino et al., 2009; Sheline et al., 2010; Oh et al., 2011; Sperling et al., 2011; Sheline and Raichle, 2013).

Functional connectivity is defined by synchronous activity of the blood-oxygen-level dependent (BOLD)-signal at spatially distant loci and indicates coupling between neuronal populations of the brain as a reflection of brain system integrity, such as cognitive processing (Friston et al., 1993; Biswal et al., 1995; van de Ven et al., 2004; Fingelkurts and Kahkonen, 2005; Buckner et al., 2008; Van Dijk et al., 2010). Assessment of functional connectivity has been performed by applying both independent component analysis (ICA) (Calhoun et al., 2001; Beckmann et al., 2005; Greicius et al., 2007; Stevens et al., 2009) and seed-driven approaches (Biswal et al., 1995; Greicius et al., 2003; Fox et al., 2005; Castellanos et al., 2008; Whitfield-Gabrieli et al., 2009; Chai et al., 2012). Seed-driven functional connectivity analysis may delineate disease-related changes in brain systems of interest based on the functional topography (Margulies et al., 2007; Cohen et al., 2008; Di Martino et al., 2008; Roy et al., 2009; Uddin et al., 2010). An integrated approach of assessing seed-based functional connectivity and component-based reduction of noise (Behzadi et al., 2007) allowing second-level random-effect analysis has been published recently (Whitfield-Gabrieli and Nieto-Castanon, 2012).

For the cerebellum, a distinct functional topography has been shown, reflecting connections to distributed circuits within the central nervous system (Stoodley and Schmahmann, 2010; Stoodley et al., 2012). These include loops associated with cognitive processing where the cerebellum exerts a modulating effect that may be impaired in neuropsychiatric disease (Middleton and Strick, 1994; Schmahmann, 1996, 1998; Schmahmann and Caplan, 2006; Schmahmann et al., 2007) and have been shown to functionally interact with intrinsic cerebral network activity (Habas et al., 2009). While coupling within various neural networks and brain systems is known to be affected by AD pathology (Buckner et al., 2008; Sheline et al., 2010; Chhatwal and Sperling, 2012; Matthews et al., 2013), to our knowledge no data has been published so far regarding potential effects of brain amyloidosis on functional integrity of the cerebro-cerebellar system.

Based on these earlier reports, we hypothesized that coupling within the cerebro-cerebellar system would be altered as a function of brain amyloidosis in elderly subjects with normal cognitive performance. Furthermore, brain amyloidosis-related changes in cerebro-cerebellar coupling may indicate cerebral brain regions with particular vulnerability to Aβ-associated pathology.

To answer this question, the current study assessed cortical Aβ load in 15 cognitively normal elderly adults by estimating cortical Pittsburgh compound B (PiB)-retention based on (11)carbon-labeled Pittsburgh compound B positron-emission-tomography (PiB-PET) late frame signals (Mathis et al., 2003; Klunk et al., 2004). In addition, resting state functional magnetic resonance imaging (fMRI) at high field strength of 7 T was performed to acquire information on regional basal neuronal activity over time at high signal-to-noise ratio (SNR). Cerebral brain regions with significant effects of cortical Aβ load on cerebro-cerebellar coupling were identified by applying second-level random-effect analysis (Whitfield-Gabrieli and Nieto-Castanon, 2012).

## Materials and Methods

### Study population

A total of 15 cognitively healthy elderly subjects without major medical or neuropsychiatric co-morbidity were recruited at the Division of Psychiatry Research and Psychogeriatric Medicine, University of Zürich, Switzerland as approved by the cantonal ethics committees of canton Zürich, Switzerland and Swiss Federal Institute of Technology, respectively (ETH Zürich) in concordance with the declaration of Helsinki (World Medical Association, 1991). Cognitive healthy status of all participants was ascertained by psychiatric examination and neuropsychological testing including mini mental state examination (MMSE) as an initial screen for cognitive impairment (MMSE) (Folstein et al., 1975). In addition, verbal learning and memory test (VLMT) (Helmstaedter and Durwen, 1990; Helmstaedter, 2001) as a modified German version of the auditory VLMT (Lezak, 1983; Müller et al., 1997) was used to assess memory performance (immediate, delayed, and supported recall), Boston Naming Test (BNT) for confrontational word retrieval (Nicholas et al., 1988), memory span [digits forward and backward for short term memory assessment (Gregoire and van der Linden, 1997)], Trail Making Test [ratio of A and B for assessment of mental flexibility (Tombaugh, 2004)] were administered. Body mass index (BMI) was assessed as a general indicator of health and calculated as reported earlier (MacKay, 2010). Exclusion criteria were: cognitive deficits indicating presence of mild cognitive impairment (MCI) or dementia (Petersen et al., 1999; Winblad et al., 2004; Albert et al., 2011), significant medication, or drug abuse that may affect cognition, general magnetic resonance imaging (MRI) exclusion criteria, contraindications against vein puncture, clinically relevant changes in red blood cell count, allergy to the Carbon-11-based PiB positron-emission-tomography (PET) tracer or any of its constituents, history of severe allergic reactions to drugs or allergens, serious medical or neuropsychiatric illness, and significant exposure to radiation, respectively.

### Generation of (11)carbon-labeled Pittsburgh compound B tracer for PET

For cerebral measures of Aβ, (11)carbon-labeled PiB-PET tracer was generated as described earlier (Klunk et al., 2004). In brief, first 11CO2C was generated using the 14N(*p*,α)11C nuclear reaction on the PET tracer cyclotron (16.5 MeV, GE) of the PET Center of the Division of Nuclear Medicine, Zürich University Hospital and processed to [11C]-methyl triflate using a AgOTf/C column at 190°C. By radiolabeling [11C]-methyl triflate with the free amine precursor 6-HO-BTA-0, the PiB-tracer was produced (Solbach et al., 2005) at 99% radiochemical purity.

### PET acquisition for assessment of cortical PiB retention

For the current study, PET scans were performed at the PET Center of the Division of Nuclear Medicine, Zürich University Hospital (GE Discovery RX STE PET/CT scanner). An individual dose equaling 350 MBq of (11)carbon-labeled PiB was applied using intravenous access to the cubital vein. PET acquisition was dynamic over 70 min, cerebral amyloid deposition was estimated based on late frame signals representing 50–70 min. The emission was corrected for attenuation (CT-based), scatter, randoms, and dead time, and reconstructed using a three dimensional (3D) Fourier re-binning (FORE) filtered back projection (FBP) algorithm, resulting in a 128 × 128 × 47 matrix with 2.34 mm × 2.34 mm × 3.27 mm voxel spacing. As a single measure of individual cortical Aβ load, cortical PiB retention scores (cortical PiB) were calculated as reported earlier (Jack et al., 2008; Vandenberghe et al., 2010; Villemagne et al., 2011). In brief, a composite score was calculated using merged cortical PiB-PET intensity values which were referenced to cerebellar intensity after co-registration applying PMOD brain tool (PNEURO) software, Version 3.4 (PMOD Technologies Ltd., Zürich, Switzerland) and transformed to standardized *Z*-scores.

### Acquisition of MRI data

Magnetic resonance imaging was performed at the Institute for Biomedical Engineering University of Zurich and ETH Zurich, Switzerland using a Philips 7-T Achieva whole-body scanner (Philips Healthcare, Cleveland, OH, USA) equipped with a nova medical quadrature transmit head coil and 32-channel receive coil array (Nova Medical, Wilmington, MA, USA). A high quality T1-weighted 3D magnetization-prepared rapid gradient-echo (MPRAGE) structural brain image covering cerebrum and cerebellum (TE/TR = 3.74/8.12 ms; resolution: 0.9 mm × 0.9 mm × 0.9 mm; total scan time: 10 min 54 s; FOV: 220 mm × 160 mm × 200 mm) was acquired for volumetric analysis of brain structures and to define cerebellar lobes used as seeds for estimation of cerebro-cerebellar coupling. Echo planar imaging (EPI)-based BOLD fMRI covering cerebrum and cerebellum (TE/TR = 25 ms/2500 ms; resolution: 1.72 mm × 1.72 mm × 3 mm; total scan time: 8 min 58 s; FOV: 220 mm × 150 mm × 220 mm) was used to acquire resting state data, suited for functional connectivity analysis.

### Post acquisition image analysis and quantification of functional connectivity

Determination of anatomical structures and estimation of volumes was performed by applying the automated image-quantification functions provided by the FreeSurfer image analysis suite (Fischl et al., 2004), using standard operations for construction of cortical models and further data analysis^1^. For improvement of functional SNR and preparation for statistical analysis, fMRI data was preprocessed using MatLab (Version 8.2 for 64-bit processors, MathWorks Inc., Natick, MA, USA) in combination with statistical parametric mapping (SPM, Version 8)^2^ (Friston, 1995) and connectivity toolbox (Conn, Version 13g)^3^ (Whitfield-Gabrieli and Nieto-Castanon, 2012). In brief, individual fMRI volumes were corrected for timing differences between slices, realigned to a reference image (B-spline interpolation based on first image of fMRI data), spatially normalized to Montreal Neurological Institute (MNI) standardized space^4^, corrected for temporal autocorrelation and spatially smoothed using a 6-mm^3^ full-width half-maximum Gaussian kernel. As described earlier, we used the art-tool^5^ to explore resting state fMRI data for artifacts and to generate a matrix of outlier-time points, which were used as first-level covariates for estimation of cerebro-cerebellar functional connectivity maps (Whitfield-Gabrieli and Nieto-Castanon, 2012). To increase specificity for gray matter signals and to reduce impact of physiological noise such as white matter and cerebrospinal fluid signals, a bandpass filter (0.01–0.1 Hz) and the anatomical component-based noise correction method (CompCor) (Behzadi et al., 2007) was applied as described earlier for seed-based functional connectivity analysis (Chai et al., 2011).

Ten bilateral region of interests (ROIs) consistent with reports on regional functional topography (Stoodley et al., 2012) were identified using automated anatomical labeling (AAL) based on the 3D MRI atlas of the human cerebellum (Schmahmann et al., 1999; Tzourio-Mazoyer et al., 2002). Mean BOLD activity within each of these ROIs was estimated and used for definition of 10 “seeds” (Joel et al., 2011): seed #1 was chosen as a representation of cerebellar lobules I–V, including the region between precentral and primary cerebellar fissure. Seed #2 includes hemispheric regions between primary and superior posterior cerebellar fissure and represents lobule VI. Seed #3 represents crus 1 and includes the hemispheric regions between superior posterior and horizontal cerebellar fissures. Seed #4 represents crus 2 and includes hemispheric regions between horizontal and ansoparamedian fissures. Seed #5 represents lobule VIIb and includes the region between ansoparamedian and prebiventer cerebellar fissure. Seed #6 represents lobule VIII and includes the region between prebiventer and secondary fissure. Seed #7 represents lobule IX and includes the region between secondary and posterolateral fissure. Seed #8 represents lobule X and includes cerebellar hemispheres caudal of the posterolateral fissure. The connectivity toolbox (Whitfield-Gabrieli and Nieto-Castanon, 2012) was used to first generate functional connectivity maps for each of the 10 cerebellar seeds, representing cerebral voxels with significant BOLD synchrony. Potential effects of cortical PiB retention (as a measure of cortical Aβ deposition) on cerebro-cerebellar coupling were tested by assessing significant second-level effects on the voxel-level using a bivariate regression approach. Adjustment of significance levels for multiple comparisons were performed as suggested earlier (Chai et al., 2011) by applying a height threshold of *p* = 0.001 and false discovery rate (FDR) correction for α = 0.05 for cluster extent as implemented in the connectivity toolbox software package (Benjamini and Hochberg, 1995; Whitfield-Gabrieli and Nieto-Castanon, 2012). Size of clusters and included brain regions were determined using the xjView-toolbox^6^ and anatomical data as provided by the AAL 3D MRI atlas of the human brain (Tzourio-Mazoyer et al., 2002). MatLab statistical toolbox (Version 8.3, MathWorks Inc., Natick, MA, USA) was used for testing hypotheses on group differences by applying unpaired Student’s *t*-test (*p*) and hypotheses on statistical dependence by applying Pearson correlation analysis (*r, p*).

## Results

### Neuropsychological testing indicates normal cognitive performance levels in the study population

Mean [standard deviation (SD)] values for MMSE were 29.47 (0.92); VLMT (immediate, delayed, and supported recall, respectively) were 11.40 (2.23), 10.80 (2.46), 12.13 (1.96); BNT were 14.73 (0.59); memory span digits forward 7.47 (1.06), digits backward (1.61); and Trail Making Test ratio A by B: 2.21 (0.66). No significant relationships between age and neuropsychological test performance scores could be observed, as indicated by Pearson correlation analysis (Table 1). Moreover, mean age was 70 years (SD 5 years), mean time of education was 14.8 years (SD 2.11). Mean BMI was 25.63 (SD 3.84), significant medical illness could be excluded based on information gathered during medical history.

**Table 1:** **Demographics of the studied sample including neuropsychological test results**.

	Mean (SD)	Correlation age	
		*r*	*p*
*N* (females/males)	15 (7/8)	–	–
Age (years)	68 (5)	–	–
Education (years)	14.8 (2.11)	0.33	0.22
Body mass index (BMI)	25.63 (3.84)	0.18	0.53
Cortical PiB retention	1.24 (0.33)	−0.42	0.12
Mini mental state examination (MMSE)	29.47 (0.92)	0.1	0.73
VMLT: immediate recall	11.40 (2.23)	0.05	0.86
VMLT: delayed recall	10.80 (2.46)	0.16	0.57
VMLT: supported recall	12.13 (1.96)	−0.31	0.26
Boston Naming Test (BNT)	14.73 (0.59)	−0.25	0.37
Memory span, digits forward	7.47 (1.06)	0.07	0.8
Memory span, digits backward	6.8 (1.61)	−0.06	0.82
Trail Making Test (ratio TMT-A by TMT-B)	2.21 (0.66)	−0.34	0.22

*Indicated are mean values with standard deviations (SD), as well as Pearson correlation coefficients (*r*) as a measure of relationship with age. *p*-Values indicate a significant relationship at *p* < 0.05*.

### Higher age is associated with increased volumes of brain stem and right amygdala in individuals with normal cognitive performance

Sequence-independent segmentation of magnetic resonance images was applied for determination of anatomical structures and estimation of regional volumes as described earlier for cerebral and cerebellar structures (Fischl et al., 2004). Pearson correlation coefficients were calculated as indicators of statistically significant relationship between regional volumes with cortical Aβ (as inferred by cortical PiB retention) and age, respectively. A relationship between age and volume that was significant when no correction for multiple testing was performed, could be observed for the brain stem (*r* = 0.53, *p* = 0.04) and right amygdala (*r* = 0.68, *p* = 0.01). No significant relationships could be observed between PiB retention and volumes in our study population of elderly individuals with normal cognitive performance (Table 2).

**Table 2:** **Volumes of brain structures as derived from the 7-T T1 MPRAGE images**.

Brain region	Volumes (ml)	Correlation PiB		Correlation age	
	Mean (SD)	*r*	*p*	*r*	*p*
Left-lateral-ventricle	19.1 (7)	0.2	0.48	0.5	0.06
Left-cerebellum-white-matter	14.1 (4.6)	0.05	0.85	0.32	0.25
Left-cerebellum-cortex	35.9 (8)	−0.02	0.95	0.02	0.94
Left-thalamus-proper	6.2 (3.8)	−0.07	0.81	0.1	0.73
Left-caudate	2.4 (0.5)	−0.19	0.49	0.09	0.76
Left-putamen	4.9 (0.6)	0	0.99	0.1	0.72
Left-pallidum	1.5 (0.2)	0.19	0.5	0.01	0.96
Third-ventricle	3.2 (2.5)	−0.13	0.65	0.21	0.45
Fourth-ventricle	2.5 (0.5)	−0.09	0.74	0.39	0.15
Brain stem	19.7 (2.2)	−0.19	0.5	**0.53**	0.04*
Left hippocampus	2.8 (1.7)	0.21	0.45	−0.11	0.69
Left-amygdala	1.1 (0.2)	−0.09	0.74	0.29	0.3
CSF	1.8 (0.8)	−0.1	0.72	0.19	0.5
Right-lateral-ventricle	19.7 (7.2)	0.03	0.93	0.43	0.11
Right-cerebellum-white-matter	13.2 (5.2)	0.23	0.41	0.48	0.07
Right-cerebellum-cortex	35.8 (5.2)	0.09	0.74	0.28	0.32
Right-thalamus-proper	6 (2.1)	−0.15	0.58	0.21	0.46
Right-caudate	3.2 (1.8)	−0.13	0.64	0.16	0.57
Right-putamen	4.6 (1.4)	−0.12	0.66	−0.16	0.56
Right-pallidum	1.3 (0.3)	0.19	0.5	−0.06	0.82
Right hippocampus	2.3 (1)	0.3	0.27	−0.33	0.22
Right amygdala	1.3 (0.4)	−0.22	0.43	**0.68**	0.01*

Indicated are mean values with standard deviations (SD), as well as correlations with age and cortical PiB [Pearson correlation coefficients (*r*), significant relationships at *p* < 0.05 are indicated by “*”]. Bold font indicates significant relationships.

### BOLD synchrony between cerebellar seeds and cerebral gray matter indicates significant cerebro-cerebellar coupling

**Figure 1:**
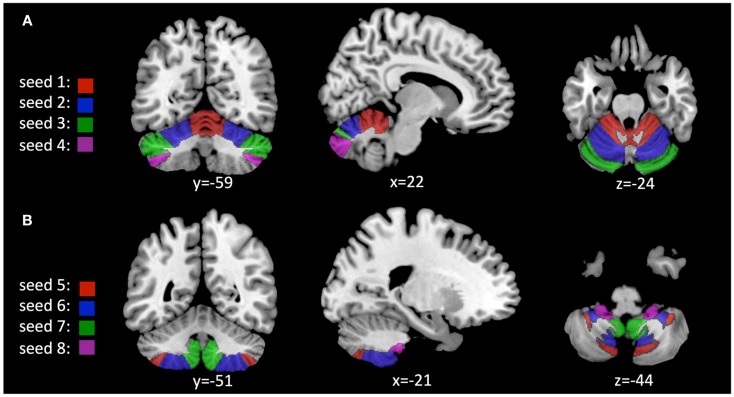
**Location of seeds used for analysis of cerebro-cerebellar coupling as derived from the AAL 3D MRI atlas (Schmahmann et al., 1999; Tzourio-Mazoyer et al., 2002): (A) Seeds #1–4, representing cerebellar lobules I–V; VI; crus 1; crus 2**. **(B)** Seeds #5–8, representing lobules VIIb; VIII; IX; X.

**Figure 2:**
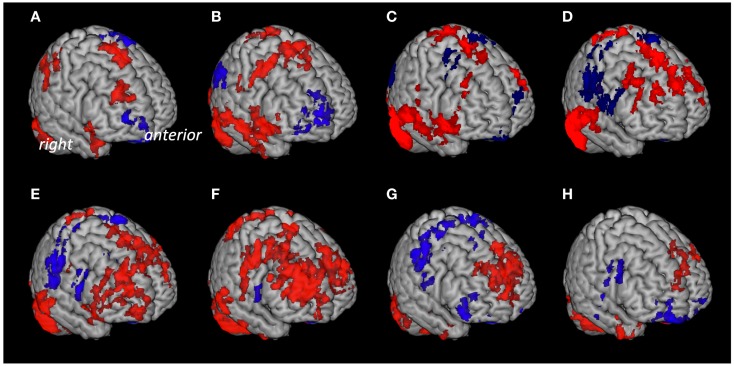
**Cerebral voxels with significant coupling to the respective seeds at rest**. Areas with “positive” connectivity are indicated in red, “negative” connectivity in blue: **(A)** Seed #1 (cerebellar lobules I–V), **(B)** seed #2 (lobule VI), **(C)** seed #3 (crus 1), **(D)** seed #4 (crus 2), **(E)** seed #5 (lobule VIIb), **(F)** seed #6 (lobule VIII), **(G)** seed #7 (lobule IX), **(H)** seed #8 (lobule X).

Based on anatomical data provided by the 3D MRI atlas of the human cerebellum (Schmahmann et al., 1999; Tzourio-Mazoyer et al., 2002), eight ROIs were generated. The ROI used for seed #1 representing cerebellar lobules I–V included a volume of 22,406 voxels; seed #2 representing lobule VI included 23,988 voxels; seed #3 representing crus 1 included 35,584 voxels; seed #4 representing crus 2 included 25,747 voxels; seed #5 representing lobule VIIb included 13,236 voxels; seed #6 representing lobule VIII included 24,420 voxels; seed #7 representing lobule IX included 9538 voxels; Seed #8 representing lobule X included 1835 voxels (Figure 1). The connectivity toolbox (Whitfield-Gabrieli and Nieto-Castanon, 2012) was used to identify the extent of BOLD synchrony between each of the eight cerebellar ROIs and cerebral voxels applying a seed to voxel approach. At a height threshold of *p* = 0.001 and cluster extent threshold of *p* = 0.05 (FDR-corrected), significant coupling between cerebellar seeds and cerebral cortical and subcortical structures could be observed as indicated by the AAL-atlas (Tzourio-Mazoyer et al., 2002) (Figure 2): for seed #1 (representing cerebellar lobules I–V) significant positive BOLD synchrony could be observed for a cerebral brain area of 47,891 voxels including occipital, temporal, limbic, and parietal lobes. Significant negative BOLD synchrony could be observed for 37,563 voxels located in the brain stem, pons, medulla, and fusiform gyrus. For seed #2 (representing lobule VI), significant positive BOLD synchrony could be observed for 46,349 voxels in the temporal, occipital, and parietal lobes. Significant negative BOLD synchrony could be observed for 45,619 voxels in the limbic lobe, uncus, medulla, and brain stem. For seed #3 (representing crus 1), significant positive BOLD synchrony could be observed for 45,554 voxels in the temporal, occipital, and parietal lobes. Significant negative BOLD synchrony could be observed for 43,099 voxels in the limbic, lobe, uncus, parahippocampal gyrus, and frontal lobes. For seed #4 (representing crus 2), significant positive BOLD synchrony could be observed for 34,463 voxels including the temporal, occipital, frontal lobes, and middle temporal gyrus, respectively. Significant negative BOLD synchrony could be observed for 34,452 voxels in the brain regions temporal lobe, Brodmann area 20, and parietal and limbic lobes. For seed #5 (representing lobule VIIb), significant positive BOLD synchrony could be observed for 32,646 voxels located in temporal, occipital frontal lobes, and pons. Significant negative BOLD synchrony could be observed for 32,943 voxels including frontal, limbic lobes, sub-lobar regions, and the parahippocampal gyrus. For seed #6 (representing lobule VIII), significant positive BOLD synchrony could be observed for 37,445 voxels including the brain regions’ temporal lobe, superior frontal gyrus, Brodmann area 9, and Brodmann area 10. Significant negative BOLD synchrony could be observed for 37,382 voxels in the limbic lobe, parahippocampal gyrus, putamen, and temporal lobe. For seed #7 (representing lobule IX), significant positive BOLD synchrony could be observed for 27,462 voxels in the temporal, occipital, and limbic lobes as well as parahippocampal gyrus. Significant negative BOLD synchrony could be observed for 26,263 voxels in frontal lobe, putamen, Brodmann area 11, and Brodmann area 47. For seed #8 (representing lobule X), significant positive BOLD synchrony could be observed for 22,907 voxels in the temporal lobe, pons, brain stem, and limbic lobe. Significant negative BOLD synchrony could be observed for 22,348 voxels in the frontal lobe, Brodmann area 11, limbic lobe, and anterior cingulate. Mean (SD) voxel size of clusters with positive BOLD synchrony was 36,840 (9213) and for negative BOLD synchrony 34,959 (7852). There was no significant difference in size between clusters of positive and negative BOLD synchrony (*p* = 0.67).

### Cortical Aβ is associated with impaired efficiency of cerebro-cerebellar coupling

**Figure 3:**
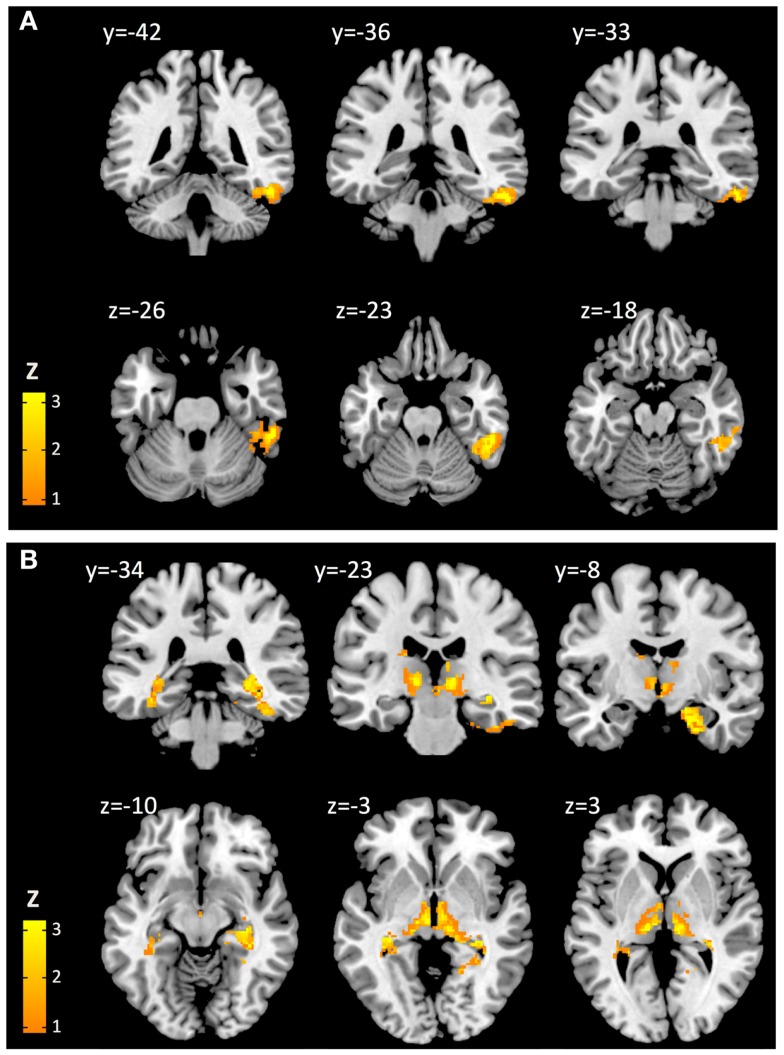
**Cerebral voxels (MNI) with significant positive effects of cortical Aβ on cerebro-cerebellar coupling for seeds representing cerebellar lobe VI (A) and lobe X (B), respectively**.

At a height threshold of *p* = 0.001 and cluster extent threshold of *p* = 0.05 (FDR-corrected), cerebral voxels with significant positive second-level effects of cortical Aβ (as estimated by cortical PiB retention) on coupling with cerebellar seeds could be observed for seed #2 (lobule VI) and seed #8 (lobule X), indicating increased BOLD synchrony as a correlate of cortical Aβ. No cerebral voxels with significant negative effects of cortical Aβ could be observed. Clusters with significant cerebral voxels were extracted using algorithms provided by the connectivity tool box (Whitfield-Gabrieli and Nieto-Castanon, 2012) and anatomically categorized based on information provided by the AAL-atlas (Tzourio-Mazoyer et al., 2002). Effects of cortical Aβ (as estimated by cortical PiB retention) on coupling with seed #2 (lobule VI) were observable for a unilateral cluster of 426 voxels with a peak at MNI: 52, −38, −24. Brain regions included, as indicated by the AAL MRI atlas (Tzourio-Mazoyer et al., 2002), were primarily located in inferior and lateral surfaces of the right temporal lobe (359 voxels), including fusiform gyrus (178 voxels), inferior temporal gyrus (126 voxels), middle temporal gyrus (53 voxels). Effects of cortical Aβ on coupling with seed #8 (lobule X) were observable for a bilateral cluster including 2205 voxels with a peak at MNI: 24, −12, −28. This cluster included voxels primarily located in the limbic (535 voxels) and temporal lobes (487 voxels). As indicated by the AAL MRI atlas, voxels were located in the following brain regions: right hippocampus (335 voxels), right parahippocampal gyrus (290 voxels), right thalamus (244 voxels), left thalamus (244 voxels), midbrain (240 voxels), left parahippocampal gyrus (173 voxels), right brain stem (148 voxels), left brain stem (91 voxels), left hippocampus (89 voxels) (Figure 3). No cerebral voxels with significant effects of age on BOLD synchrony could be detected in our sample when applying a height threshold of *p* = 0.001 and FDR-corrected cluster extent threshold of *p* = 0.05.

## Discussion

By performing resting state fMRI at high field strength of 7 T and assessment of cortical Aβ by PiB-PET in a sample of cognitively normal elderly subjects, a significant effect of cortical Aβ on cerebro-cerebellar coupling was observable. Our data indicate decreased network efficiency that particularly affects brain regions located in the limbic lobe, temporal lobe, and subcortical gray nuclei including hippocampus, parahippocampal gyrus, fusiform gyrus, inferior temporal gyrus, and thalamus. No effect of cortical Aβ on volumes of brain regions was observable, which appears consistent with the high level of cognitive performance measured in the study sample and also earlier considerations that functional alterations may precede brain atrophy in Aβ-associated neurodegenerative illness such as AD.

All participants included in the current study displayed normal cognitive performance levels, as indicated by neuropsychological testing including MMSE (Folstein et al., 1975), VLMT (Helmstaedter and Durwen, 1990), BNT (Nicholas et al., 1988), memory span (Gregoire and van der Linden, 1997), and mental flexibility (Tombaugh, 2004). In our sample, no relationship between lower test performance and higher age was observable, indicating absence of aging-related cognitive decline in the studied sample.

Pittsburgh compound B positron-emission-tomography (Mathis et al., 2003; Klunk et al., 2004) is the most extensively studied and best validated tracer for quantification of Aβ accumulation in the human brain (Rabinovici and Jagust, 2009) and was used in the current study to assess the degree of individual cerebral Aβ load in all study participants. While cerebral Aβ deposition is a hallmark of AD (Alzheimer, 1907; Hock and Nitsch, 2000; Albert et al., 2011), recent studies applying PiB-PET have demonstrated an association of individual Aβ load with increased risk for aging-related cognitive decline and incidence of AD, respectively (Jagust et al., 2009; Sperling et al., 2011; Shaffer et al., 2013; Wirth et al., 2013). To obtain a single measure of individual cortical Aβ load, we calculated cortical PiB retention scores (cortical PiB), indicating global cortical uptake of the PiB-PET tracer normalized to intensity of the cerebellum (Jack et al., 2008; Vandenberghe et al., 2010; Villemagne et al., 2011).

Processing of cognitive tasks has been shown to be associated with distinct neuronal systems whose activity is reflected by several functional networks of spatially distant brain regions, linked by synchronous BOLD activity both under challenge as well as at rest (Biswal et al., 1995; Woodward et al., 2006; Buckner et al., 2008; Fair et al., 2009). While integrity of functional brain networks has been shown to be impaired in the context of many neuropsychiatric illnesses (Calhoun et al., 2008; Buckner et al., 2009; Whitfield-Gabrieli and Ford, 2012; Unschuld et al., 2013a,b), there is a concatenation of studies that indicate cerebral Aβ accumulation as a significant interference factor on physiological brain network activity (Woodward et al., 2006; Hedden et al., 2009; Sheline et al., 2010; Oh et al., 2011). However, while there are publications reporting effects of Aβ on integrity of many brain networks, to our knowledge there are no studies published that investigate effects of Aβ on cerebro-cerebellar coupling.

For this study, resting state fMRI at high field strength of 7 T was used for assessment of basal neural activity at increased SNRs, which has been demonstrated to provide increased sensitivity for BOLD activity and assessment of intrinsic network activity compared to lower field strength MRI (Donahue et al., 2010, 2011; Lenglet et al., 2012; Theysohn et al., 2013; Zhan et al., 2013). To measure functional connectivity within the cerebro-cerebellar system and its potential alteration in a context of cortical Aβ load, as indicated by cortical PiB retention scores, a bivariate regression analysis was applied using algorithms provided by the connectivity toolbox (Whitfield-Gabrieli and Nieto-Castanon, 2012). While earlier studies investigating functional connectivity in the cerebro-cerebellar system used both ICA (Habas et al., 2009) as well as seed-based approaches (He et al., 2004; Allen et al., 2005), we chose a seed-based approach as it allows specific investigation of functional brain systems and produces results that are consistent with ICA experiments (Joel et al., 2011). Based on the AAL 3D MRI atlas (Schmahmann et al., 1999; Tzourio-Mazoyer et al., 2002) and earlier information on functional topography of the cerebellum (Stoodley et al., 2010, 2012), eight cerebellar ROI were chosen as seeds. As brain atrophy is a frequent finding associated with aging (Scahill et al., 2003) and also has been shown to relate to Aβ load in AD (de Leon et al., 1997; de Leon and Klunk, 2006; Storandt et al., 2009), volumes of cortical and subcortical brain regions were assessed by applying the FreeSurfer analysis suite (Fischl et al., 2002, 2004) and then tested for relationship with cortical Aβ. To be able to distinguish effects due to cortical Aβ from effects attributable to other factors associated with aging, also relationship between cerebro-cerebellar coupling and brain volumes, respectively, with age was tested.

Our data are consistent with earlier reports as a significant degree of functional connectivity could be observed between cerebellar seeds and cerebral voxels, many of whom are attributable to well-established functional networks (Allen et al., 2005; Habas et al., 2009). However, connectivity maps in the current dataset involve structures that diverge from earlier assumed roles for the respective cerebellar subregions (Stoodley and Schmahmann, 2009; Stoodley et al., 2012). This may be explained by the fact that earlier studies have applied task-paradigms for mapping cerebellar cognitive processing, whereas we assessed basal neural activity at rest. In addition, as our data has been acquired at high magnetic field strength of 7 T, the fact that additional network activity may be detected due to increased SNRs needs to be considered when comparing network properties to studies performed at lower field strength as discussed earlier (Zhan et al., 2013). Moreover, our data indicate a significant effect of cortical PiB on cerebro-cerebellar coupling that is observable for seeds representing lobule VI and X. While lobule VI has been shown earlier to be involved in executive function as well as salience processing (Allen et al., 2005; Stoodley and Schmahmann, 2009), to our knowledge so far no fMRI studies inferring a functional role for cerebellar lobule X have been published. However, the absence of motor syndrome after ischemic infarction of the flocculonodular lobe may indicate relevance of lobule X for non-motor tasks and cognitive processing (Schmahmann et al., 2009). We could identify cerebral voxels with an increase of coupling associated with higher cortical PiB in brain regions including hippocampus, parahippocampal gyrus, fusiform gyrus, and inferior temporal gyrus but also thalamus. While all of these brain regions have been shown to be affected by atrophy in a context of AD and brain Aβ deposition (Bakker et al., 2008; Jack et al., 2008; Storandt et al., 2009; Ballard et al., 2011), our finding of increased connectivity might be a reflection of AD pathology-associated brain change, resulting in reduced network efficiency. Moreover, the here described effects of cortical Aβ may indicate increased vulnerability of the cerebro-cerebellar system, consistent with earlier considerations on neuronal changes in presymptomatic AD (Mormino et al., 2009; Jagust, 2013). As tasks carried out by the cerebro-cerebellar system include cognitive processes such as visual-spatial, executive function, and working-memory (Schmahmann and Pandya, 2008; Stoodley, 2012), which frequently are affected in subjects diagnosed with MCI or AD (Hock and Nitsch, 2000; Storandt et al., 2009; Albert et al., 2011; Ballard et al., 2011), prospective longitudinal studies may reveal the impact of cerebro-cerebellar dysfunction on cognitive dysfunction in AD. The fact that we find effects of Aβ on cerebro-cerebellar connectivity but no relationship between Aβ and volumes may be consistent with earlier reports on functional changes potentially preceding brain atrophy in presymptomatic AD (Sperling et al., 2009; Sheline et al., 2010; Sheline and Raichle, 2013). However, we do find a nominally significant association of higher brain stem volume and amygdala volume with age, which may reflect resilience for maintaining a normal level of cognitive functioning at higher age and potentially interacts with other neuronal factors of cognitive reserve (Stern, 2009; Steffener and Stern, 2012). As *p*-values indicating significance of this relationship were not corrected for multiple comparisons, this finding on potential relevance of brain stem and amygdala volume needs to be treated with caution. While we do not find an effect of aging on cerebro-cerebellar coupling, our data does not provide evidence against the possibility that there nevertheless may exist a relationship between higher age and functionality of the cerebro-cerebellar system that might be observable in a larger sample that represents a wider age range of study participants. Moreover, recent data derived both from animal and neuropathological studies indicates that several pathological processes may take place in parallel to or as an upstream event of brain Aβ accumulation (Gruart et al., 2008; Krstic and Knuesel, 2013). While the experimental setup of our study only allowed to assess effects of Aβ load on cortico-cerebellar coupling, potential modulating effects reflecting, e.g., tauopathy or regional sublevel inflammation thus could not be taken into account.

Taken together, by using *in vivo* PiB-PET imaging, we could demonstrate that high levels of cortical Aβ are associated with aberrant cortico-cerebellar coupling in cognitively normal elderly individuals. Cerebral regions particularly affected by altered coupling are consistent with established patterns of increased sensitivity for pathological brain change in AD. These findings suggest that Aβ is linked to functional reorganization within the cerebro-cerebellar system, taking place in elderly individuals with normal cognitive performance. Additional longitudinal studies including patients with MCI and AD are necessary to figure out up to what degree Aβ-associated alterations in the cerebro-cerebellar system contribute to clinical symptoms or potentially represent an early stage of AD with increased risk for progression to dementia.

## Conflict of Interest Statement

The authors declare that the research was conducted in the absence of any commercial or financial relationships that could be construed as a potential conflict of interest.

## Footnotes

^1^http://surfer.nmr.mgh.harvard.edu/

^2^http://www.fil.ion.ucl.ac.uk/spm/

^3^http://www.nitrc.org/projects/conn/

^4^http://www.mni.mcgill.ca/

^5^http://www.nitrc.org/projects/artifact_detect

^6^www.alivelearn.net/xjview8/xjview.m
